# Multifocal structured illumination optoacoustic microscopy

**DOI:** 10.1038/s41377-020-00390-9

**Published:** 2020-08-31

**Authors:** Zhenyue Chen, Ali Özbek, Johannes Rebling, Quanyu Zhou, Xosé Luís Deán-Ben, Daniel Razansky

**Affiliations:** Institute for Biomedical Engineering and Institute of Pharmacology and Toxicology, University of Zurich and ETH Zurich, Zurich, Switzerland

**Keywords:** Microscopy, Imaging and sensing

## Abstract

Optoacoustic (OA) imaging has the capacity to effectively bridge the gap between macroscopic and microscopic realms in biological imaging. High-resolution OA microscopy has so far been performed via point-by-point scanning with a focused laser beam, thus greatly restricting the achievable imaging speed and/or field of view. Herein we introduce multifocal structured illumination OA microscopy (MSIOAM) that attains real-time 3D imaging speeds. For this purpose, the excitation laser beam is shaped to a grid of focused spots at the tissue surface by means of a beamsplitting diffraction grating and a condenser and is then scanned with an acousto-optic deflector operating at kHz rates. In both phantom and in vivo mouse experiments, a 10 mm wide volumetric field of view was imaged with 15 Hz frame rate at 28 μm spatial resolution. The proposed method is expected to greatly aid in biological investigations of dynamic functional, kinetic, and metabolic processes across multiple scales.

## Optoacoustics: Real-time microscopic imaging gets onto the grid

Microvascular structures inside living tissues can now be imaged at rates suitable for video playback. Daniel Razansky from the University and ETH Zurich in Switzerland and colleagues have improved the speed of optoacoustic microscopes that use laser-generated ultrasonic waves to visualize subcutaneous tissue. Creating clear 3D microscopic images from optoacoustic data normally requires sequential focusing onto different points on a sample. The Swiss-based team reports that splitting the laser beam into a grid of multiple focused spots enables parallel data collection. The grid, produced with a diffraction grating and a two-lens condenser, can operate at multiple spatial scales. Calibration experiments and trials with live mice revealed the new microscope could offer a large centimetre-scale field of view at high speeds while simultaneously capturing microscopic details.

## Introduction

A myriad of light excitation and ultrasound detection embodiments have been proposed for optoacoustic (OA) interrogation of biological tissues at scales ranging from sub-cellular structures to whole rodents and selected parts of the human body^1,2^. Image formation methods have been accordingly devised to provide spatial resolutions that typically scale with 1/200 of the imaging depth^3–5^. For depths within the transport mean free path of photons (~1 mm in biological tissues), the resolution can be established optically by shaping the excitation light beam. Optical-resolution OA microscopy (OR-OAM) has thus facilitated microvascular imaging with a typical resolution of a few microns^6^, which can be further enhanced beyond the optical diffraction limit at the very surface of the sample^7,8^. On the other hand, OA can be adapted to provide ultrasonic resolution at millimetre to centimetre depths, where the excitation light beam is fully diffuse^9,10^. In particular, OA tomography (OAT) can accurately resolve the optical absorption distribution from ultrasound (pressure) signals acquired at a sufficient number of locations enclosing the imaged region. State-of-the-art OAT systems based on spherical matrix arrays capitalize on the simultaneous excitation of a tissue volume, parallel acquisition hardware, graphics processing unit-based data processing and fast wavelength-tuning lasers to accelerate the acquisition and visualization of volumetric spectrally resolved OA data to frame rates on the order of tens to hundreds of Hz^11–15^. Sparse signal acquisition combined with total-variation-based reconstruction has further enabled boosting of imaging speeds to an unprecedented kHz range in full three dimensions (3D), thus enabling a unique capability to monitor ultrafast biological phenomena in entire volumes at sub-millisecond time scales^13^. While OR-OAM systems have been significantly accelerated with respect to the initially reported implementations^16,17^, their basic point-by-point scanning approach has remained unaltered for most OR-OAM systems. This has imposed significant limitations on the achievable temporal resolution and/or field of view (FOV), thus greatly restricting the range of potential applications especially when it comes to imaging of rapid biological dynamics.

To overcome these limitations, several approaches have been explored to facilitate faster OAM image acquisition. Acceleration of the scanning speed has been achieved by using lasers with higher pulse repetition frequencies (PRFs) and rapid beam steering with mirrors mounted on piezo scanners or microelectromechanical systems (MEMS). This approach enables ultrafast scanning rates but is limited to relatively small FOVs, typically far below 1 mm^18^. A larger FOV can be achieved through mechanical steering of both the optical and ultrasound fields, e.g., through physical scanning of the entire ultrasound detector and light-guiding elements with piezo or voice-coil stages^19–21^, albeit at the expense of generally slower imaging rates. Water-immersed scanning optical mirrors have similarly enabled acoustic beam deflection and effective scanning of the sensitivity field of a focused ultrasound detector. It is, for instance, possible to achieve very fast B-scan rates across FOVs of several millimetres by means of a one-dimensional (1D) galvo mirror immersed in non-conducting liquid hydrofluoroether^22^ or an immersible hexagon-mirror scanner driven by a DC motor^23^. Alternatively, small optical sensors with broad angular acoustic sensitivity have been suggested in which a larger FOV can be achieved solely by scanning the optical beam^24^. While state-of-the-art OR-OAM systems have reached a B-scan rate of 900 Hz over a 1 mm scanning range, it still takes 8 s to render a high-resolution image over a 10 × 10 mm² FOV with a scanning step of 20 μm^23^.

An alternative solution for accelerating OR-OAM is based on acquiring data from multiple excitation spots instead of increasing the PRF of the laser and the associated beam scanning speed. A multifocal imaging system consisting of a linear arrangement of 20 microlenses combined with a linear ultrasonic array has been shown to increase the imaging speed by 20-fold compared to a single-point scanning strategy^25^. However, sample scanning is still necessary to avoid confocal misalignment, making the system impractical for in vivo imaging applications. An alternative solution has been developed based on scanning a microlens array parallel to the cross-sectional imaging plane of a full-ring ultrasonic transducer array^26^. However, the imaging speed was ultimately limited by the mechanical scanning speed and the throughput capacity of the data acquisition electronics to 36 s per 10 × 10 mm² FOV.

**Fig. 1:**
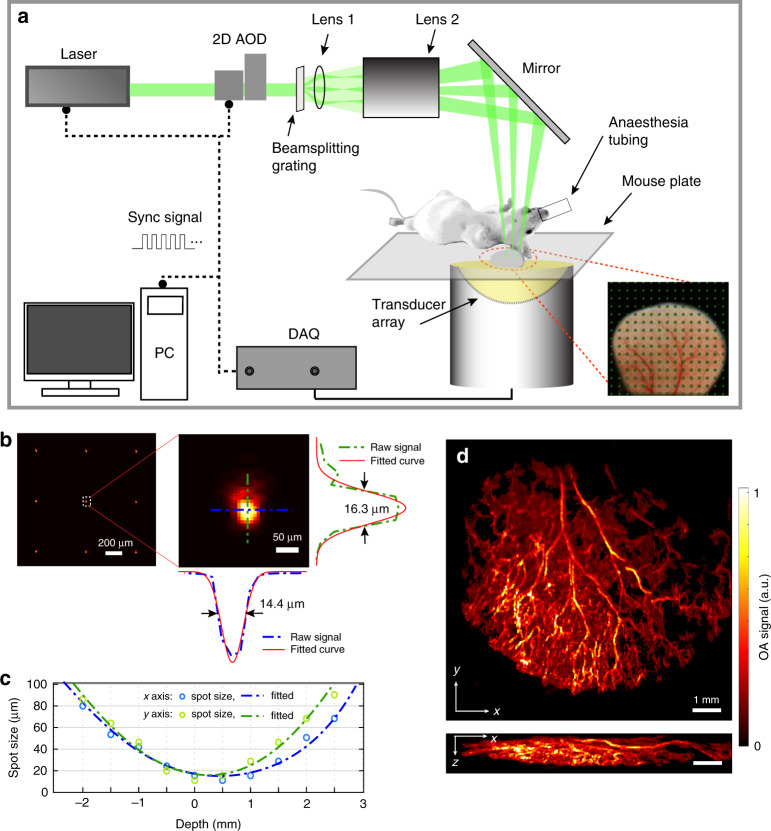
Multifocal structured illumination optoacoustic microscopy (MSIOAM) method and system characterization. **a** Layout of the experimental system. **b** Part of the illumination grid measured with a beam profiler and one magnified spot along with its horizontal and vertical profiles. **c** Measured beam size along the axial light propagation direction. **d** Exemplary 3D image of an entire mouse ear obtained via MSIOAM. Maximum intensity projections (MIPs) along the *z* and *y* axes are shown

Herein, we propose a new multifocal structured illumination OA microscopy (MSIOAM) approach for scalable imaging with high resolution and speed. The multifocal pattern is generated by a beamsplitting grating and a two-lens condenser and is combined with parallel signal acquisition by means of a spherical matrix array transducer (Fig. 1a). Images are subsequently formed by calculating (reconstructing) the signal intensities of the individual spots corresponding to the intersections of the light beams and microvascular structures. Owing to its 3D tomographic acquisition geometry, the suggested system is not solely limited to superficial optical-resolution imaging but can additionally operate at multiple penetration scales by gradually exchanging microscopic optical resolution in superficial tissues for ultrasonic resolution at greater depths, where the light becomes more diffuse.

## Results

The developed MSIOAM system is schematically depicted in Fig. 1a and described in detail in the “Materials and methods” section. For in vivo demonstration, a mouse ear was imaged, as shown in the photograph. We employed a nanosecond-duration laser beam for OA excitation, which was shaped to a grid of spots at the tissue surface by means of a beamsplitting diffraction grating and a condenser composed of two lenses. Figure 1b displays part of the beam pattern at the focal plane as measured with a beam profiler (SP620u, Ophir Optronics, USA) along with a zoomed-in view of one of the optical foci and the corresponding horizontal and vertical beam profiles. According to an image of the illumination grid provided by the manufacturer, it has good uniformity with a ±4.9% intensity distribution (Supplementary Fig. S1). We also imaged a black-painted microscope slide, for which we recorded fluctuations in the ±8% range for the OA signals detected from the different foci, i.e., much lower than the signal changes induced by actual samples (Supplementary Fig. S1). Note that the spot size, the distance between adjacent spots and the depth of field (DOF) are all dependent on the effective size of the incident laser beam and the working distance and thus can be adjusted accordingly. In the suggested configuration, these parameters can easily be adjusted by modifying the working distance of Lens 2 to find the optimal trade-off between the spot size and DOF. For the given configuration, the measured sizes of the individual spots remained below 20 μm for a DOF of more than 1 mm (Fig. 1c). Image formation in MSIOAM is based on the 3D localization of isolated sources excited with the structured light beam (see the “Materials and methods” section for details). Accordingly, the light spot size at the tissue surface determines the achievable lateral resolution, while the axial resolution is associated with the ability to localize the individual spots generating the OA responses. An exemplary image of a mouse ear obtained via MSIOAM is shown in Fig. 1d.

**Fig. 2:**
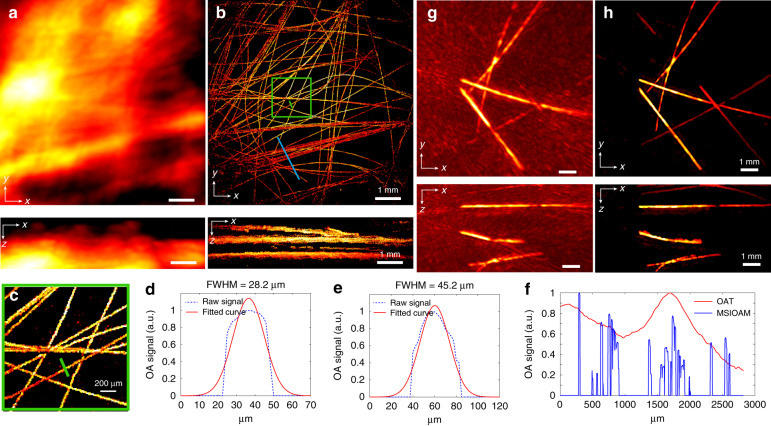
Performance characterization results based on 3D phantoms. **a** Volumetric image of a phantom containing randomly arranged carbon fibres of 7 µm in diameter obtained via standard OAT with broad illumination of the sample and spherical array detection—maximal intensity projections (MIPs) along the *z* and *y* directions are shown. **b** The corresponding MSIOAM image obtained with multifocal structured illumination. **c** Zoomed-in image of the region indicated by the green box in **b**. **d**, **e** Lateral and axial signal profiles along the green line in **c**, together with the corresponding Gaussian fitted curves. **f** Signal profile comparison of the OAT and MSIOAM images along the blue line in **b**. **g**, **h** OAT and MSIOAM images of a phantom containing randomly arranged human hairs

To comprehensively characterize the effective spatial resolution of the system, a phantom containing 7 μm diameter carbon fibres was used. The superior resolution and image quality achieved with MSIOAM are clearly visible in the images (Fig. 2). While the fibres forming a cluster appear completely indistinguishable in the OAT images acquired using broad (unfocused) illumination (Fig. 2a), they can be clearly discerned via MSIOAM (Fig. 2b, c). The measured full-width-at-half-maximum (FWHM) of the image profiles in the lateral and axial directions are 28 and 45 μm, respectively (Fig. 2d, e). Signal profiles extracted from the OAT and MSIOAM images along the blue line marked in Fig. 2b are also compared to further illustrate the clear enhancement of the lateral resolution achieved with MSIOAM (Fig. 2f). Note that this lateral resolution enhancement is chiefly associated with the signal-to-noise ratio (SNR) of the individual spots^27^, while the inferior axial resolution can be partially attributed to ultrasonic diffraction due to the lack of optical focusing along the axial dimension.

The imaging performance of MSIOAM was also tested using a larger phantom containing a random arrangement of black human hairs. A comparison between the images obtained via MSIOAM and regular OAT is shown in Fig. 2g, h. Note that both the OAT images, corresponding to broad sample illumination, as well as the individual frames used to form the final MSIOAM image were reconstructed using a 3D back-projection (BP) algorithm^28^. The weak scattering of light by the agar and the large depth of focus of the illumination pattern facilitated high-resolution MSIOAM imaging across an effective depth range of ~5 mm without additional axial scanning. These findings illustrate the unique capabilities of this approach for the large-scale microscopic imaging of samples with arbitrarily curved surfaces. The real-time preview displayed during data acquisition is shown in Supplementary Video 1.

**Fig. 3:**
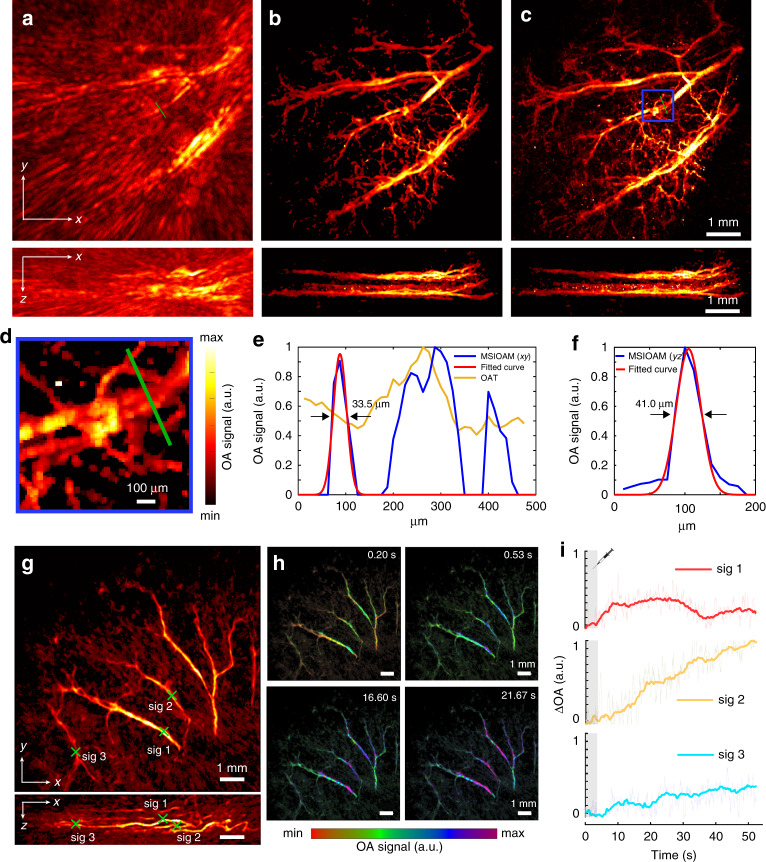
In vivo mouse ear imaging results. **a** Example of an OAT image of a mouse ear acquired using broad illumination—maximum intensity projections (MIPs) along the *z* and *y* axes are shown. **b** The corresponding MSIOAM image reconstructed from 50 × 50 scanning frames with all 512 channels recorded simultaneously. **c** Averaging 10 consecutive MSIOAM frames has a positive effect on the resulting image quality and resolution due to the increased SNR. **d** Zoomed-in view of the area indicated by the blue box in panel **c**. **e** Signal profile comparison of the OAT and MSIOAM images along the green line in panel **d**. **f** Corresponding signal profile along the axial direction. **g** MSIOAM image of a mouse ear acquired via 10 × 10 sparse sampling with 128 random channels—MIPs along the *z* and *y* axes are shown. **h** Colour-coded OA images representing four different time points during the perfusion of 25 nm diameter gold nanoparticles through the vascular network. The data were recorded at an effective frame rate of 15 Hz for the finally reconstructed MSIOAM images. **i** Temporal signal profiles from the three regions of interest (ROIs) indicated in panel **g**

We further characterized the in vivo imaging performance of MSIOAM for the mouse ear by also directly comparing it to regular OAT. The relatively low detection bandwidth of the array sensing elements hinders the visualization of microvascular structures when merely performing standard OAT measurements with uniform illumination of the entire sample (Fig. 3a). In contrast, when the structured illumination approach is employed, the locations of the individual spots in the vascular network can be readily discerned in the images reconstructed from a single scanning position (Supplementary Fig. S2). The multifocal illumination grid was then scanned with a very dense pattern consisting of 50 × 50 positions over the mouse ear, and all 512 channels were recorded for each laser pulse with the laser PRF set to 200 Hz, resulting in a 12.5 s acquisition time for the combined MSIOAM image (Fig. 3b). The equivalent image obtained by averaging the signals from 10 scans of the optical grid is shown in Fig. 3c. Such averaging enhances the SNR of the images of individual spots and hence increases the localization accuracy, ultimately leading to higher resolution at the cost of a longer acquisition time. Note that the lateral size of the smallest resolvable vessels was ~34 µm for the MSIOAM image (Fig. 3c) versus ~200 µm in the OAT image (Fig. 3a), corresponding to a sixfold improvement in the achievable lateral resolution. This is further illustrated by the zoomed-in image (Fig. 3d) as well as the 1D signal profiles extracted from the OAT and MSIOAM images in the transverse plane (Fig. 3e) and sagittal plane (Fig. 3f). A movie showing rotating 3D views of the reconstructed images is available in the online version (Supplementary Video 2).

The image acquisition process was subsequently accelerated by reducing the scanning pattern to 10 × 10 positions and increasing the laser PRF to 1.5 kHz. Due to the limited 1 Gbps output bandwidth of the data acquisition system, the number of simultaneously recorded channels was also reduced to 128, thus resulting in sparse tomographic data. However, reasonable image quality could still be obtained (Fig. 3g). More importantly, it was possible to accelerate the effective frame rate to 15 Hz, i.e., a 67 ms image acquisition time. The real-time imaging capability was further demonstrated by visualizing the perfusion of gold nanoparticles through the mouse ear. Due to the strong absorption of light by blood at 532 nm, only subtle, yet clearly detectable, signal variations can be observed in the time-lapse image sequence (Fig. 3h). Differential signal profiles, calculated by subtracting the pre-injection signal values from the three regions of interest (ROIs) indicated in Fig. 3g, are also shown in Fig. 3i (normalized to the maximum value). A signal increase associated with agent perfusion through the selected vessels can be clearly observed in these normalized profiles, while further revealing different perfusion dynamics in the imaged vessels. The real-time dynamics of the contrast agent are best visualized in a movie available in the online version of the journal (Supplementary Video 3).

Finally, much like in conventional scanning OAM, the spatial resolution and overall image quality of MSIOAM will naturally deteriorate with increasing imaging depth within a scattering medium. To illustrate this effect, a phantom consisting of a microscope slide painted black was imaged through scattering layers of various thicknesses consisting of 0.6% intralipid (v/v) dissolved in agar. As expected, the SNR of the OA images decays with increasing depth (Supplementary Fig. S3). While the SNR can be improved by signal averaging, the increase in the spot size due to light diffusion within the turbid medium cannot be rectified by averaging, leading to diminished resolution performance (Supplementary Fig. S3).

## Discussion

Herein, we report on the development of MSIOAM as a new method to overcome the imaging speed limitations of conventional OAM techniques. The new approach has been shown to attain high (optical) resolution, a centimetre-scale FOV and real-time volumetric imaging rates, a combination of metrics unachievable with the existing OAM or OAT embodiments. The highly parallelized data acquisition protocol in MSIOAM enables coverage of a large FOV with a significantly higher temporal resolution than conventional OAM methods based on mechanical or optical scanning of a single laser spot. For the latter, the scanning speed is restricted by the performance of the mechanical stage and/or scanning mirrors, while the trade-off between the FOV and imaging speed is also inherently limited by the ultrasound time of flight. For example, a conventional OAM system employing an objective with a high numerical aperture (NA) can achieve submicron lateral resolution at the expense of a very small FOV and a diminished DOF, thus hindering 3D imaging^29–32^. In contrast, the low NA and large DOF of the focused diffracted beams used for signal excitation in MSIOAM facilitate the imaging of large and curved tissue surfaces such as the rodent brain^29^. Since MSIOAM involves no mechanical scanning of either the transducer or the light beam, this represents an important advantage over previously reported OAM parallelization attempts employing microlenses or linear ultrasound arrays^25,26^. The real-time imaging performance has critical implications for the visualization of dynamic biological phenomena, e.g., those associated with perfusion, neural activity, breathing or other rapid in vivo motion. The latter constitute a considerable challenge for conventional OAM approaches, in which millions of scanning positions are required to render one high-resolution image.

The MSIOAM approach enables a suitable compromise among key imaging performance characteristics. While the diameter of the individual light spots at the tissue surface is sufficiently small to resolve microvascular structures, such as arterioles or venules, the resolution could further be enhanced to the detriment of the DOF. The particular MSIOAM image formation approach also greatly improves the image contrast since only signals generated by the excitation foci eventually contribute to the reconstructed images. The lateral resolution is determined by the spots’ size and their localization accuracy while the sparsity of the generated acoustic signals is fully determined by the illumination grid. Similar to other OAM and OAT methods, the axial resolution in MSIOAM is generally determined by the acoustic diffraction parameters, i.e., by factors such as the transducer bandwidth and angular tomographic coverage^27^.

It should be noted that the imaging speed of MSIOAM is ultimately limited by the 1 Gbps bandwidth of the data acquisition system. Nevertheless, multifocal illumination and parallel data acquisition facilitate rapid data collection, further relaxing the beam scanning speed requirements. In contrast, scanners based on galvo mirrors or other types of mirrors, including piezo and MEMS devices, are fundamentally limited by the inertia associated with the mass of the rotating mirror and other moving parts. This is particularly true for scanners with a large mirror size (e.g., 5 mm in diameter). Although the imaging speed of MSIOAM can be further accelerated by increasing the throughput capacity of the data transmission interfaces, the currently achieved performance is adequate for many relevant biological applications involving, e.g., haemodynamics, brain activity, agent perfusion and uptake or monitoring responses to treatments^10,11^.

Several important aspects and limitations need to be considered when designing an optimal MSIOAM system. Volumetric OA imaging with spherical arrays is known to benefit from their large angular coverage to enable accurate tomographic OA reconstructions^33,34^. Furthermore, in a spherical array geometry, all the individual elements are directed towards the centre of the FOV, thus facilitating higher sensitivity and better OA image quality compared with standard (e.g., linear or planar) array transducers. However, an inherent trade-off exists between the array characteristics, i.e. its detection bandwidth, number and arrangement of elements, etc., and the resulting image quality and speed. On the other hand, due to the small NA of each mini-beam, the smallest achievable spot size is usually larger than in other OAM methods employing a single focused beam, making MSIOAM unsuitable for imaging on a single micrometre resolution level. Compared to other parallelized OAM techniques incorporating microlens arrays, no mechanically translated parts exist in MSIOAM, and the spot size and FOV can be readily adjusted. Compared to OAM systems employing spatial light modulators (SLMs) or digital micro-mirror devices (DMDs), beamsplitting gratings have a much higher diffraction efficiency, making them more suitable for the multifocal illumination strategy. Furthermore, the grid pattern generated by a DMD or SLM usually needs to be demagnified prior to projecting it onto the sample, resulting in a very small (submillimetre) FOV^35^. In our proposed approach, the scanning of the illumination grid is implemented by applying a series of acoustic frequencies to the AOD. The laser beam is then deflected to the corresponding angular positions with a scanning frequency of up to the hundred-kilohertz level. Although AODs have a smaller deflection angle range (typically <0.05 rad) than mirror-based mechanical deflectors, they can provide a significantly larger active optical window (up to 10 mm) with a much higher maximum deflection velocity (up to 2.5 × 10^5^ rad/s) and deflection angle accuracy («0.1 μrad)^36,37^. In our current implementation, the maximum required deflection angle range corresponds to the inter-angle between different diffraction orders of the grating, i.e., 9.9 mrad, which is much smaller than the achievable deflection angle of an AOD. Taking all of the above factors into consideration, the combination of an AOD and a beamsplitting grating is mutually reinforcing and thus ideal for fast scanning microscopy applications. These optimization measures are expected to enable a broad range of applications well beyond what is currently feasible with OA imaging systems, especially applications involving the investigation of fast biodynamics with mesoscopic-scale resolution.

A number of further developments are foreseen for the MSIOAM concept. The current results are based on OA images reconstructed from densely sampled signals. Recently, it has been shown that a combination of sparse data sampling and a reconstruction framework based on total variation regularization in the spatial and temporal domains enables the accurate rendering of OA images while reducing the amount of data by a factor of 16 ^13^. The development of parallel acquisition electronics operating at faster frame rates in the kHz range along with new algorithms properly accounting for the spatio-temporal sparsity of the acquired sequence of OA images could thus significantly boost the temporal resolution of MSIOAM. Moreover, it is possible to perform OA imaging with a single detector by capitalizing on the complex propagation of ultrasound waves^38–40^. On the other hand, the functional and molecular imaging potential of OA imaging can be enhanced by exciting biological tissues at multiple optical wavelengths, thus enabling the mapping of the bio-distributions of substances featuring specific optical absorption spectra^41,42^. Accordingly, a multispectral MSIOAM system can potentially be developed. It has been shown that multilayer diffractive optical elements can achieve broadband beam diffraction with a uniform energy distribution and high efficiency^43,44^. By combining two beamsplitting gratings with two different dispersive materials and suitable phase patterns, chromatic aberration can be corrected, thus enabling multispectral OA imaging with wideband light sources or different laser sources simultaneously. However, such an effort would face challenges related to the availability of laser sources with sufficient energy and PRF over a relatively wide spectral range.

Importantly, in the current implementation, the excitation light beam is transmitted from the opposite side of the spherical array (trans-illumination mode), which may restrict the range of potential applications. An epi-illumination (reflection mode) arrangement is potentially feasible considering that the spherical transducer array used for OA signal detection features a cylindrical aperture for excitation light transmission. Such an aperture has recently been exploited for hybrid OA and epi-fluorescence imaging^45^ and could potentially be designed to include the optical path for MSIOAM. The development of MSIOAM in an epi-illumination configuration may further enhance its scalability and mitigate some acoustic distortions arising from wave propagation through heterogeneous tissues^46,47^. In this regard, the use of spherical arrays for ultrasound detection in volumetric OAT has recently been shown to attain unprecedented spatio-temporal resolution performance and a scalable penetration depth into diffuse tissues^10^. Accordingly, the MSIOAM approach could potentially be extended for imaging in both optical (in superficial tissues) and acoustic (at diffuse light depths) resolution modes using the same spherical detection array. A dedicated image reconstruction approach would be essential for such an implementation, especially to achieve accurate reconstruction in the mesoscopic transition region where the individual mini-beams are not fully diffused^48,49^.

In conclusion, we have proposed a highly parallelized imaging method based on multifocal structured illumination and a spherical array detector that can effectively bridge the microscopic and macroscopic realms in OA imaging. By means of rapid scanning of the grid-like illumination pattern, fast microscopic OA imaging in optical-resolution mode can be achieved with a centimetre-scale FOV. The proposed method is expected to facilitate biological studies investigating fast functional and dynamic processes, such as neuroimaging, contrast agent uptake, organ perfusion or cell tracking.

## Materials and methods

### Imaging system

Schematic diagram of the proposed MSIOAM system is presented in Fig. 1. A frequency-doubled Q-switched diode-pumped Nd:YAG laser (model: IS8II-E, EdgeWave, Germany) operating at 532 nm wavelength is used for OA signal excitation, with a PRF and energy per pulse that are adjustable up to 10 kHz and 3 mJ, respectively. The laser beam is raster scanned with a two-dimensional acousto-optic deflector (2D AOD) (AA Opto-Electronic, France) and guided into a customized beamsplitting (diffraction) grating (Holoeye GmbH, Germany) to split the light energy into an equally spaced grid of beams. For a grating with 15 × 15 diffraction orders, the angular separation is 0.57° with a diffraction efficiency of ~74% and a uniformity of intensity within 10% for all diffraction orders. The angle between adjacent diffraction modes is substantially smaller than the maximum scanning range of the AOD (2.292°). The diffracted light beams are focused by two condensing lenses (Lens 1 and Lens 2 in Fig. 1, 35 mm focal length) to generate a grid of spots (optical foci) at the surface of the sample^50^. The excited OA signals are collected by a custom-made ultrasonic array detector (Imasonic SaS, France) consisting of 512 piezocomposite elements of 2.5 mm in diameter, with a 5 MHz central frequency and a ~100% detection bandwidth at FWHM. The array elements are uniformly distributed on a spherical surface with a 40 mm radius and 140° angular coverage (1.3*π* solid angle)^51^. The signals detected by the array are sampled by a 512-channel data acquisition system at 40 mega-samples per second and are then transferred to the computer via a 1 Gbps Ethernet connection. Such a spherical array has been shown to provide an almost isotropic resolution of 150–250 µm for an effective FOV of 10 × 10 × 10 mm^3^ by imaging an agar phantom containing sparsely distributed 50 μm diameter polyethylene-absorbing spheres^52^.

### System characterization

The structured light beam generated by the beamsplitting grating was first characterized by measuring the light intensity distribution at the focal plane of Lens 1 (Fig. 1) with a beam profiler (SP620u, Ophir Optronics, USA). The spot size, the distance between grid points and the DOF at the sample plane were then measured at the focal plane of Lens 2, for which the beam profiler was translated around its focal plane to acquire a sequence of images at different depths. The DOF was characterized by measuring the spot FWHM along the optical axis. The pulse energy after the deflector was measured with a power meter (EnergyMax Sensors, Coherent, USA) during the scanning process to account for the dependency of the diffraction efficiency of the 2D AOD on the incidence angle.

### Phantom experiments

Two agar (1.3% w/v) phantoms embedded with either 7 μm diameter carbon fibres (SIGRAFIL C T24-5.0, Germany) or human hairs were first imaged using the MSIOAM system. The spherical volume enclosed by the ultrasonic array was also filled with 1.3% w/v agar for optimal coupling of the acoustic signal. A 15 × 15 beamsplitting grating, 35 mm plano-convex lens (Lens 1) and 35 mm f/2D Nikon lens (Lens 2) were employed to generate the multifocal structured illumination. The measured pulse energy on the sample surface was ~120 μJ, i.e., ~0.53 μJ per illumination spot. During the experiment, the laser pulsing, AOD beam scanning and data acquisition processes were synchronized by an external function generator running at 200 Hz. For the carbon fibre phantom measurement, the illumination pattern was raster scanned over a grid of 150 × 150 positions (4 μm scanning step, 112.5 s total acquisition time), whereas the hair phantom was scanned over 25 × 25 positions (24 μm scanning step, 3.125 s total acquisition time). The OA signals were simultaneously recorded from all 512 channels of the spherical array for every frame. The spatial resolution of the MSIOAM system was characterized by measuring the FWHMs of the reconstructed carbon fibres along all Cartesian dimensions.

### Animal experiment

The performance of MSIOAM was subsequently validated in vivo by noninvasively imaging the ears of three athymic nude-Fox1nu mice (Harlan Laboratories Ltd, Switzerland). The mice were anaesthetized with isoflurane (3.0% v/v for induction and 1.5% v/v during the experiments) in 20% O_2_ and 80% air at an approximate flow rate of 1 l/min. During each experiment, the mouse was placed in the supine position on a plastic plate (with an opening), which was attached to the casing of the array detector to avoid any movement or misalignment. The mouse ear was positioned along the focal plane of the array detector to ensure good signal detection sensitivity. Ultrasound gel was applied to the mouse ear to facilitate acoustic coupling. The number of scanning grid points was varied between 50 × 50 and 10 × 10, with the scanning step accordingly adjusted between 12 and 60 μm. With these settings, we were able to accelerate the effective frame rate up to 15 Hz for the in vivo imaging experiments, i.e., a 67 ms acquisition time for the final MSIOAM image. For the perfusion monitoring experiment, a 100 µl volume containing 25 nm diameter gold nanoparticle solution (concentration of 10^15^ particles per ml) was injected through the tail vein. All procedures involving mice conformed to the national guidelines of the Swiss Federal Act on animal protection and were approved by the Cantonal Veterinary Office of Zurich.

### Image reconstruction

The image formation principle in MSIOAM is based on the localization and superposition of the OA sources^27^. First, filtered BP reconstruction^28^ was performed for each scanning position considering a 10 × 10 × 10 mm^3^ region containing 400 × 400 × 400 voxels. Second, regional maxima were sought and located within the reconstructed 3D volume. The illumination pattern information was considered as an initial guess for the positions of such regional maxima. OA sources corresponding to illumination spots on absorbing structures could be robustly distinguished from the background. Third, virtual pinholes were applied to the selected signal spots, and the surrounding voxels were zeroed out. Finally, all images in the sequence formed in the third step were superimposed to form the final image of the structure of interest. In this step, data interpolation was performed for signal superimposition depending on the distance between two adjacent illumination spots and the number of scanning steps. All processing steps were performed in MATLAB (R2019a, MathWorks, USA) using custom-developed software unless otherwise stated.

## Supplementary information

Supplementary figures

Supplementary Video 1

Supplementary Video 2

Supplementary Video 3
