# Supplementary material for: Multifocal structured illumination optoacoustic microscopy
Source: Light Sci Appl. 2020 Aug 31;9:152. doi: 10.1038/s41377-020-00390-9 (PMC7459102; doi:10.1038/s41377-020-00390-9)
Supplement: Supplementary file 1 — Supplementary figures [file 41377_2020_390_MOESM1_ESM.pdf]

Supplementary Information for

Multifocal structured illumination optoacoustic microscopy

Zhenyue Chen, Ali Özbek, Johannes Rebling, Quanyu Zhou, Xosé Luís Deán-Ben, and Daniel Razansky*

Institute for Biomedical Engineering and Institute of Pharmacology and Toxicology, University of Zurich and ETH Zurich, Switzerland

* *Corresponding author:* daniel.razansky@uzh.ch

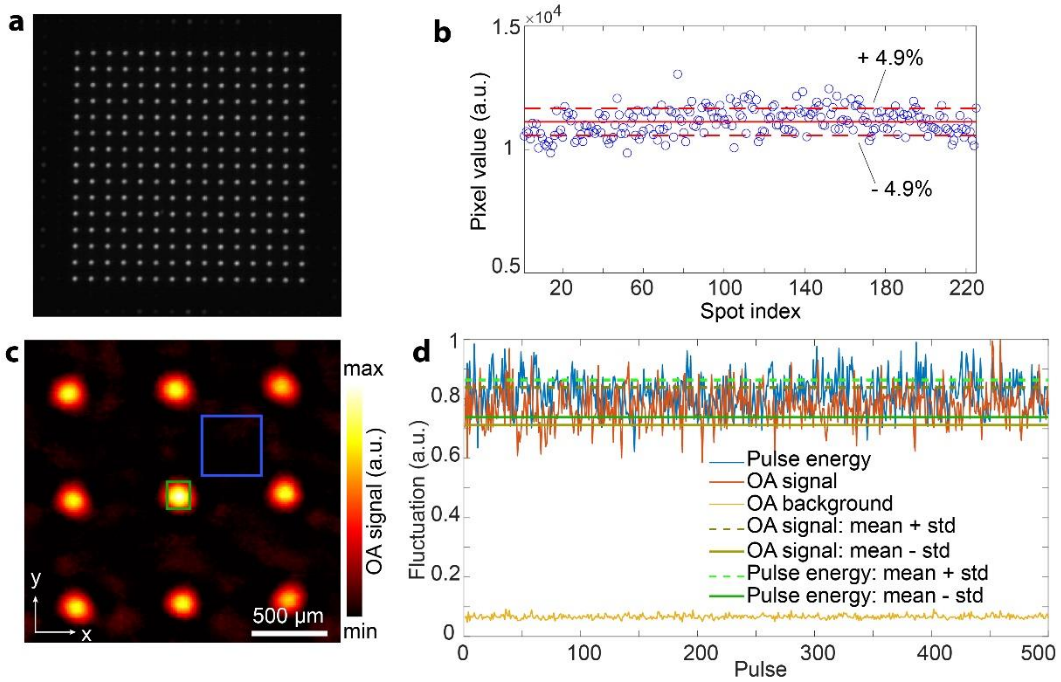

Fig. S1 Measurements of the illumination grid uniformity error and pulse to pulse fluctuations. **a** Image of the illumination grid acquired with a camera (provided by the manufacturer of the diffraction grating). **b** Mean value and standard deviation (4.9 %) of the intensity distribution of the illumination grid. As shown in the plot, most of the intensities fall within a range of ± 4.9 % surrounding the mean value. **c** Optoacoustic (OA) image of a glass slide covered with black paint with the multifocal structured illumination. MIP view along the z direction is shown. The same measurement was repeated over 500 consecutive laser pulses. **d** Pulse to pulse fluctuations of the laser pulse energy and optoacoustic signals. Laser pulse energy was measured with a powermeter (LabMax, Coherent, USA). The mean pixel values in the green box and blue box in **a** were set as the OA signal and OA background, respectively. The pulse energy fluctuation was 7.5% calculated by std(pulse energy)/mean(pulse energy). Similarly, the OA signal fluctuation was 8.1% which was in agreement with the pulse energy fluctuation. The OA background fluctuation was calculated to 10.7% due to the relatively low signal to noise ratio.

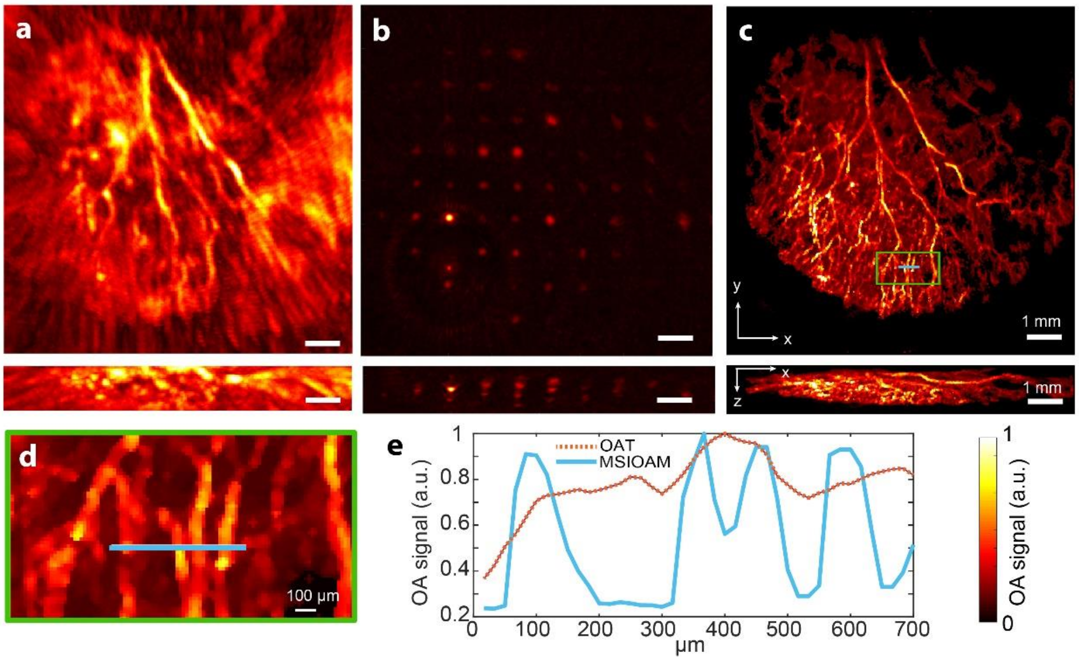

Fig. S2 *In vivo* experimental results for mouse ear imaging. **a** OAT images of the mouse ear using broad illumination – maximum intensity projections (MIPs) along z and y axis are shown. **b** Equivalent OAT image of the ear reconstructed from a single scanning position of the patterned light grid. **c** Equivalent MSIOAM image reconstructed from 50 x 50 scanning frames with all the 512 channels. **d** Zoom into the area labeled by a green box in panel **c**. **e** Signal profile comparison for the OAT and MSIOAM images along the blue line indicated in **d**.

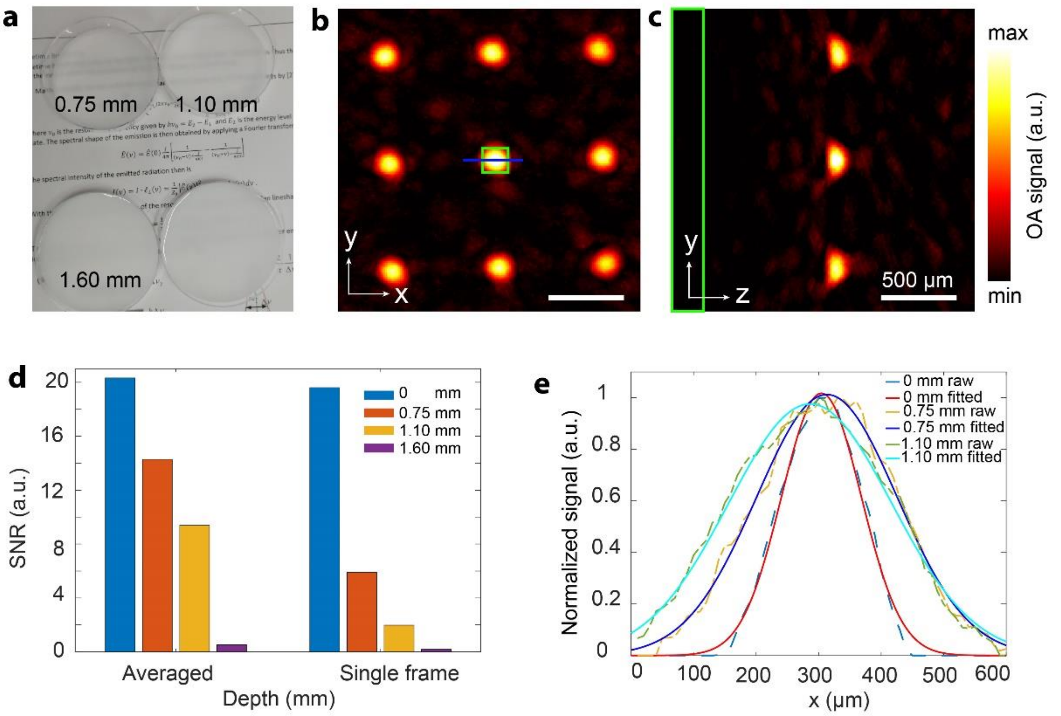

Fig. S3 Experimental measurement of SNR and spot size vs. imaging depth. **a** Tissue mimicking phantoms made with agar and 3% intralipid. Different thicknesses of the scattering layer were tested, i.e., 0.75 mm, 1.10 mm and 1.60 mm. For the measurements, the layers were placed on top of a glass slide covered with black paint. **b, c** Optoacoustic images of a piece of the slide acquired with the multifocal structured illumination. Maximum intensity projection (MIP) along the z and x directions are shown. **d** SNR values measured for the different imaging depths (agar layer thickness). The signal value was calculated by the mean pixel values in the green box indicated in **b**. The noise level was calculated by the standard deviation of voxels in the first 10 slices in the reconstructed 3D volume. On the left side, the averaged SNR was calculated in the z-axis MIP image reconstructed by averaging 1000 frames of the raw data. The corresponding measurements for single frames are shown on the right. **e** Line profiles of the signal spot at different depths along the blue line indicated in **b**. The full-width-at-half-maximum (FWHM) calculated by Gaussian fitting is 150.2 µm, 265.0 µm and 306.6 µm for tissue mimicking phantom with thickness of 0 mm, 0.75 mm and 1.10 mm, respectively.
